# Assessing the Impact of Demographic Factors on Presenting Conditions or Complaints Among Internal Medicine Patients in an Underserved Population in Central Florida

**DOI:** 10.7759/cureus.27811

**Published:** 2022-08-09

**Authors:** Angie El-Said, Rasika Patil, Brianna Leone, Aaishwariya Gulani, Matt P Abrams, Aamir Momin, Judith Simms-Cendan

**Affiliations:** 1 Medicine, University of Central Florida College of Medicine, Orlando, USA; 2 Obstetrics and Gynecology, University of Miami Miller School of Medicine, Miami, USA

**Keywords:** public health, underserved population, internal medicine, student-run clinic, demographics

## Abstract

Background and objective

Patients’ demographics (race, age, gender, and ethnicity) have been determined to affect patients’ health status. It has been established that chronic disease prevalence varies by race, age, gender, and ethnicity; however, not much is known about how these demographic factors influence presenting conditions or complaints within a student-run clinic (SRC). This study aimed to investigate how demographic factors in the Apopka community in Florida determine what internal medicine (IM) conditions or complaints patients present with at a student-run free clinic.

Methods

Electronic medical record (EMR) data for adult patients seen at the clinic from February 2019 to February 2020 were reviewed to collect information on patient demographics, IM presenting conditions or complaints, and body mass index (BMI). Binary logistic regressions were employed to investigate the relationship between demographic factors and presenting conditions or complaints.

Results

The majority of the patients were female (62.2%), with an almost equal representation of Hispanic (50.3%) and non-Hispanic individuals. About half of the patients visiting the clinic were either overweight or obese. Of the 167 patients, the average age was 44.17 and 44.32 years for males and females respectively. The most common presenting conditions or complaints included cardiac conditions (25.07%), diabetes (9.64%), gastric pain (9.21%), and upper respiratory infection (URI)/allergies (6.15%). Cardiac conditions were further broken down into hypertension (18.94%), dyslipidemia (3.94%), and palpitations (2.19%). Patient age was a contributing factor to the incidence of diabetes (p=0.002), hypertension (p<0.0001), and cardiovascular conditions excluding hypertension (p=0.021). There was a significant relationship between obesity and diabetes (p=0.036) and hypertension (p=<0.001).

Conclusion

SRCs can make use of the information obtained from this study to advocate for coverage of medications to treat diabetes and hypertension in this undocumented population to prevent morbidity rates. We believe our findings can also provide guidance in terms of instituting screening programs for these illnesses among the broader population and SRCs with different patient makeups.

## Introduction

The association of age, gender, race, and ethnicity with health status has been extensively investigated in the medical literature. More specifically, the incidence, prevalence, and management of various diseases have been studied in terms of these parameters and it has been determined that health status varies among race, age, gender, and ethnicity [1]. For example, ethnicity also disproportionally impacts population health. In 2018, a literature review highlighted that the incidence and prevalence of type 2 diabetes (T2DM) in Hispanic children, adolescents, and adults were higher than the national average [2].

Although extensive research has been performed to investigate patient demographics, a comprehensive assessment of a patient’s demographic influence on patient management remains scarce in the literature. Researchers at the Kansas City Free Eye Clinic (KCFEC), for example, characterized the average patient population receiving eyecare services at this student-run free clinic to be African American males who were homeless and uninsured [3]. This study recommended conducting further research on the types of presenting conditions or complaints most common among this population.

The Apopka Clinic, a multi-disciplinary student-run clinic (SRC) hosted by the University of Central Florida College of Medicine and the Farmworkers Association of Florida, caters to the healthcare needs of migrant farmworkers in Apopka, Florida. The clinic was established in July 2016 in collaboration with the Farmworker Association of Florida to provide health services to individuals without health insurance. By providing services to individuals with barriers to access to healthcare services, this clinic caters to an underserved population (as defined by the United States Department of Health and Human Services) mainly comprising farmworkers [4]. Services provided every quarter at the Apopka Clinic include those related to internal medicine (IM), pediatrics, ophthalmology, nephrology, obstetrics/gynecology, dermatology, and physical therapy.

A prior study that served as a model for the present study was conducted based on electronic medical records (EMR) at the Miami Rescue Mission Clinic [5]. Researchers collected data on chronic disease prevalence and sociodemographics and compared them to county and state-level statistics. Higher rates of hypertension, diabetes, obesity, asthma, and mental health issues were found among the clinic’s patient population when compared to the local population. The present study was conducted using a similar methodology to understand the demographics of Apopka patients, as well as their most common presenting conditions or complaints, and compare them to those of the greater Orlando area. Generally, SRCs assess the demographics and/or prevalence of diseases. This study is unique in that it intended to assess how a patient’s demographics such as age, gender, ethnicity, and race may influence the progression of chronic disease as reported by the patient’s conditions or complaints at an SRC.

The objective of this study is to investigate the relationship between demographics and presenting conditions or complaints among farmworkers in Apopka, FL, with a special focus on IM, and how this data compares to city, county, state, and national statistics. Providers may find such correlations useful to better understand what presenting conditions or complaints are the most common ones within these demographics, which can lead to increased efficiency and accuracy in patient management.

## Materials and methods

Study population and design

This study protocol was adapted from the study by Zhang et. al. conducted at the Miami Rescue Mission Clinic and was approved by the Institutional Review Board at the University of Central Florida (STUDY#: 0002711) [5]. This was a descriptive, retrospective review of EMR data investigating presenting conditions or complaints among adult patients who presented to a student-run free clinic from February 28, 2019, to February 27, 2020, within IM. This time period corresponds to the adoption of a new EMR and the transition from in-person clinics to telehealth due to the COVID-19 pandemic. Data were eligible for review if the patient was over 18 years of age and had at least one presenting condition or complaint related to IM. All patient information had been previously collected by medical student volunteers and overseen by medical faculty volunteers. All data were retrieved from the EMR and then transferred onto a spreadsheet by four individuals. The data retrieved included patient gender, age, race, ethnicity, body mass index (BMI), and presenting complaints, or conditions previously diagnosed by outside physicians. Each patient was de-identified by assigning them a random patient identification number. The presenting conditions or complaints were classified into nine categories [hypertension, diabetes mellitus, dyslipidemia, physical exam, upper respiratory infection (URI)/allergies, palpitations, gastric pain, vision, and “other” with respect to what patients presented with]. Hypertension, dyslipidemia, and palpitations were further combined into cardiovascular conditions. Gastric pain was defined as having a presenting complaint or condition of either abdominal pain or gastroesophageal reflux disease (GERD). To assess whether hypertension altered the significance of cardiovascular conditions, a separate regression model with only dyslipidemia and palpitations was run. Since many patients coming in with cardiac conditions were coming in with regard to hypertension, another logistic regression was run for hypertension alone. Although dyslipidemia could be grouped separately, the study's objectives included assessing cardiovascular conditions as well. Since dyslipidemia causes atherosclerotic disease, which is a component of cardiovascular disease, it was decided to group dyslipidemia within cardiac conditions.

Analysis

Multivariate binary logistic regression was carried out using demographics including age, gender, and ethnicity as categorical variables to determine how they contribute to presenting conditions or complaints within IM. To avoid collinearity within the analysis, ethnicity was chosen over race as there were more entries in the EMR for this variable. To ensure independence, only the first visit to IM was included in the analysis. Separate multivariate binary logistic regression models were run for each of the presenting conditions or complaints (cardiac conditions, diabetes mellitus, vision, URI/allergies, physical exam, hypertension, and other types of presenting conditions or complaints) to determine which demographic parameters served as risk factors. Cardiac disease, which includes palpitations, and dyslipidemia were run in two separate models with and without hypertension, to determine if hypertension would impact results. Pearson’s chi-square test of independence was performed to assess the relationship between obesity and demographics, e.g., ethnicity and gender, and presenting conditions or complaints, e.g., diabetes, cardiac disease, and hypertension. A p-value less than 0.05 was considered statistically significant. Statistical analysis was conducted using IBM SPSS Statistics version 27 (IBM Corp., Armonk, NY) [6].

## Results

Patient demographics

Of the 167 patients, the average age was 44.17 and 44.32 years for males and females respectively (Table 1). The majority of patients frequenting the clinic were female (62.2%). About 50% of the patients seen in IM were considered overweight or obese. According to the World Health Organization (WHO), a BMI between 25 kg/m^2^ and 30 kg/m^2^ is defined as overweight, and a BMI above 30 kg/m^2 ^is defined as obese [7].

**Table 1:** Patient demographic data BMI: body mass index

Variables	Values, % (N=167)
BMI, kg/m^2^	
Underweight	0%
Normal	26.9%
Overweight	18%
Obesity I	16.8%
Obesity II	10.8%
Obesity III	4.2%
Unknown	23.4%
Race	
White	24%
African American	13.2%
Hispanic	39.5%
Asian	0.6%
Puerto Rican	1.8%
Unknown	21%
Age, years	
18-39	41.3%
40-64	49.1%
65+	9.6%
Gender	
Male	37.8%
Female	62.2%

BMI of patients visiting the clinic was unknown (23.4%), underweight (0.00%), normal (26.9%), overweight (18%), obesity I (16.8%), obesity II (10.8%), and obesity III (4.2%). The ethnicity of patients visiting the clinics was Hispanic-Latino (50.3%) and non-Hispanic-Latino (49.7%). Regarding race, patients visiting the clinic were unknown (21%), White (24%), African American (13.2%), Hispanic (39.5%), Asian (0.6%), and Puerto Rican (1.8%). Of note, patients can be and have been reported as being Hispanic-Latino and White.

Prevalence of presenting conditions or complaints

A total of 167 unique patients were seen for IM services with 228 presenting conditions or complaints (Table 2). These included hypertension (18.4%), diabetes (9.64%), obesity (3.94%), dyslipidemia (3.94%), conditions requiring a physical exam (3.5%), URI/allergies (6.14%), palpitations (2.19%), gastric pain (9.21%), vision-related (3.95%), and others (39%). Conditions relating to cardiovascular disease were the most common (26.28%). The other types of presenting conditions or complaints included rashes, joint and back pain, and general symptoms like nausea and vomiting.

**Table 2:** Patient-reported presenting conditions or complaints seen at Apopka Farmworker’s Clinic Internal Medicine Service URI: upper respiratory infection

Presenting condition or complaint	Prevalence, % (N=228)
Hypertension	18.94%
Diabetes	9.64%
Obesity	3.94%
Dyslipidemia	3.94%
URI/Allergies	6.15%
Requiring physical exam	3.5%
Palpitations	2.19%
Gastric pain	9.21%
Vision-related	3.95%
Other	39%

Association between demographics and presenting conditions or complaints

Using logistic regression, it was determined that patient age served as a risk factor for diabetes (OR=1.054, 95% CI=1.019-1.090, p=0.002) as seen in Table 3, cardiovascular disease (B=0.059, OR=1.061, 95% CI=1.032-1.090, p<0.001) as seen in Table 4, hypertension (B=0.049, OR=1.050, 95% CI=1.022-1.079, p<0.001) as seen in Table 5, and cardiovascular disease excluding hypertension (B=0.045, OR=1.046, 95% CI=1.007-1.088, p=0.021) as seen in Table 6. Age, gender, and ethnicity did not contribute to the chance of a patient coming in with presenting conditions or complaints regarding vision, gastric pain, URI/allergies, and “others.” Age and gender associations were assessed for each model, and there was no statistical significance within the models.

**Table 3:** Association between diabetes and age, gender, and ethnicity Age serves as a contributor to diabetes (p=0.002) *Indicates p-value is less than 0.05 and statistically significant

Demographic	Logistic regression coefficient (B)	Odds ratio	95% confidence interval	P-value
Age	0.053	1.054	1.019-1.090	0.002*
Gender (male versus female)	0.882	0.810	0.810-7.202	0.1114
Ethnicity (non-Hispanic versus Hispanic)	0.072	0.415	0.415-2.786	0.882

**Table 4:** Association between cardiac disease and age, gender, and ethnicity Age serves as a contributor to cardiovascular disease (p=0.004) *Indicates p-value is less than 0.05 and statistically significant

Demographic	Logistic regression coefficient (B)	Odds ratio	95% confidence interval	P-value
Age	0.059	1.061	1.032-1.090	<0.001*
Gender (male versus female)	-0.338	0.713	0.345-1.474	0.361
Ethnicity (non-Hispanic versus Hispanic)	0.303	1.354	0.666-2.755	0.403

**Table 5:** Association between cardiovascular disease (excluding hypertension) and age, gender, and ethnicity Age serves as a contributor to cardiovascular disease (p=0.004) *Indicates p-value is less than 0.05 and statistically significant

Demographic	Logistic regression coefficient (B)	Odds ratio	95% confidence interval	P-value
Age	0.045	1.046	1.010-1.080	0.021*
Gender (male versus female)	1.031	2.804	0.734-10.712	0.381
Ethnicity (non-Hispanic versus Hispanic)	0.351	1.421	0.463-4.364	0.538

**Table 6:** Association between hypertension and age, gender, and ethnicity Age serves as a contributor to hypertension (p=0.000) *Indicates p-value is less than 0.05 and statistically significant

Demographic	Logistic regression coefficient (B)	Odds ratio	95% confidence interval	P-value
Age	0.049	1.050	1.022-1.079	<0.001*
Gender (male versus female)	-0.756	0.469	0.221-0.995	0.053
Ethnicity (non-Hispanic versus Hispanic)	0.224	1.251	0.592-2.642	0.576

Association of obesity with patient demographics and select presenting conditions and complaints

A Pearson's chi-square test of independence was performed to assess the relationship between obesity and demographics, e.g., ethnicity and gender, and presenting conditions or complaints, e.g., diabetes, cardiac disease, and hypertension. There was a significant association between obesity and diabetes (X2(1, N=128) = 4.386, p=0.036) and hypertension (X2(1, N=128) = 13.149, p<0.001). There was no significant relationship between obesity and cardiac disease excluding hypertension (X2(1, N=128) = 0.052, p=0.820), ethnicity (X2(1, N=128) = 0.290, p=0.590), and gender (X2(1, N=128) = 1.474, p=0.225).

## Discussion

Although Central Florida migrant farmworkers have indicated an interest in receiving more nutritional information, they often do not have the means to purchase sufficient and nutritious food [8]. Similar to what has been found among the populations served by SRCs across the United States, in this study, a large segment of patients was either overweight or obese (50%). Within this population, a majority of patient conditions were related to cardiovascular disease and more specifically hypertension (45%). A study by Luo et al. (2018) showed that while farmworkers seek out care by attending free clinics, a majority of their concerns still remain centered around social needs like finances, and their families' well-being, rather than their own health [9].

Regarding the contribution of demographic variables to patients' presenting conditions or complaints, age was the only significant risk factor as determined through this analysis. Age was determined to be a significant risk factor for general cardiac conditions, hypertension, and diabetes. However, age was not found to be a risk factor for other patient complaints or conditions including gastric pain complaints, vision complaints, allergies, and conditions requiring physical exams. This could be explained by the fact that cardiovascular diseases and diabetes are associated with chronic inflammation and worsen with poor lifestyle choices over time [7]. The other conditions are not as often linked to chronic inflammation and therefore do not correlate as highly with increasing age. As discussed previously, the farmworker patient population studied has a high prevalence of obesity. Obesity and elevated BMI as well as eating saturated fats and sodium for many years can lead to an accumulation of damage and inflammation in the body [10-11]. In general, BMI, age, diet, and T2DM are some of the major risk factors that contribute to the increased incidence of cardiovascular disease [11]. Within this population, an elevated BMI has a significant association with hypertension and diabetes, as shown in prior literature, but not with cardiac disease excluding hypertension, ethnicity, and gender [12].

The chief complaints of many patients attending student-run free clinics are similar to those in our unique population; thus, our findings provide additional evidence of the types of conditions that students at SRCs need to know as to how to educate and treat their patients. That is, the data highlight that even though students in this study may be working with various different populations, the issues the patients at this SRC face are generally those related to chronic noncommunicable diseases (NCDs). These are rising rapidly in both developing countries and the United States [13]. While not all SRCs treat immigrants, many do, thereby making this study generalizable to other SRCs in states with a high influx of immigrants such as California, Texas, and Florida. Previously, education and intervention strategies for patients at SRCs were often aimed at poor hygiene, limited clean water, etc. [14]. NCDs are difficult to address in any population as recent studies have shown that education is less effective at changing behavior than implementing changes in local policies and laws [15]. SRCs can use the information gained from this study as additional evidence to advocate for coverage of medications to treat diabetes and hypertension in this underserved population to prevent morbidity rates. A better understanding of this population can also guide instituting screening programs for these illnesses among the broader population and SRCs with different patient makeups. These findings have already helped determine what type of further care these patients need (for example, subsequently seeking out ophthalmology care to look for the sequelae of hypertension and diabetic retinopathy).

It is well known that cardiovascular disease, hypertension, and diabetes have a high prevalence in the general population. However, some models of SRCs do not serve patients longitudinally while some do offer long-term care. Additionally, there are SRCs that only focus on one type of care, while other clinics offer their services for several conditions. To ensure effective care for the patient, the understanding that some clinics have different profiles of patients will help provide more focused care. In an SRC on the Mexico-Texas border, the most common presenting conditions were also diabetes, hypertension, and dyslipidemia [16]. Similarly, a study published regarding an SRC in Puebla, Mexico came to the same conclusion [17]. In Northern Ontario, diabetes, cardiovascular disease, and mental illness were among the most prevalent in terms of needs assessment [18]. Within different locations, needs assessments often remain the same. This study, therefore, highlights the parallels among various clinics and the need to implement adequate screenings and long-term care for common NCDs [19]. This study suggests that there are perhaps no significant risk factors among patients at this SRC aside from age for NCDs; however, other clinics can try similar strategies to assess what serves as risk factors in their communities and implement respective screenings and develop a model of care centered around long-term care.

Although SRCs have slightly different models, several of the presenting conditions among patients within a student-run free clinic are similar, which highlights the relevance of this study for all SRCs. It reinforces the importance of SRCs assessing their own patient demographics and determining the types of patients their common presentations are associated with.

How the leading health profiles of the Apopka Clinic patients compare to those of the larger city, state, and national demographics remains a point of interest (Table 7). Although patients that present to the Apopka Clinic often have similar presenting conditions or complaints to other SRCs, as mentioned before, the prevalence of reported conditions or complaints falls below the city, state, and national average. This study relied heavily on patients' presenting complaints or conditions. Barriers to accessing healthcare secondary to time and cost, language barriers, and decreased health literacy may potentially serve as confounding variables not accounted for, which may have led to a lower prevalence of patient-reported complaints and conditions in our study compared to diagnosed conditions within the wider United States population. Another explanation can be the relatively small sample size of 167 patients, compared to a much larger general population. If patients do not report these conditions more often, this may also serve as a confounding variable, which could explain why gender and ethnicity were not found to be risk factors in this study.

**Table 7:** Classification of the data related to obesity, diabetes, high blood pressure, and high cholesterol among Apopka farmworkers against the county, state, and country data The demographics of obesity, diabetes, high blood pressure, and high cholesterol broken down by data from this study against the county, state, and national data. For obesity, diabetes, high blood pressure, and high cholesterol, the prevalence was determined only by the presenting conditions or complaints patients presented with during their encounters at the clinic [20-24].

	Apopka farmworkers	Apopka	Florida	USA
Obesity	31.8%	38.10%	37.80%	42.4%
Diabetes	9.64%	9.20%	10.70%	10.5%
High blood pressure	18.14%	20.50%	25.10%	47%
High cholesterol	2.19%	16.70%	20.70%	38%

Our study has several limitations. The use of the current EMR began on February 28, 2019, limiting the number of patients that could be included. Patients came in with several unique presenting conditions or complaints that did not fit under one major category, and hence these unique presenting conditions or complaints were allocated as “other”. With a larger patient population, the nine presenting conditions or complaints that were recognized might have been different. Over 20% of patients in this study did not indicate their ethnicity. Due to this lack of data, we could not determine how ethnicity correlated with presenting conditions or complaints in about a fifth of the sample. All data were collected and coded manually. This manual entry of data is another limitation since human errors can lead to reduced precision and accuracy of variables collected and analyzed. However, an effort was made to review the entries prior to the analysis.

In the future, reassessment of the migrant farmworker community demographics based on EMR data of a longer period may be possible. By increasing the number of unique patients in the study, their presenting conditions or complaints can be better categorized, and the use of the “other” category can be removed. Additionally, investigating the contribution of access to nutrition has on obesity, diabetes, and high blood pressure would be of interest.

## Conclusions

Within the migrant farmworker community in Apopka, FL, there is a high number of patients who present with cardiovascular disease and/or diabetes. There is also a high prevalence of obesity in this underserved population, which highlights the need for more proactive interventions by the healthcare workers in this clinic. These interventions may include acquiring and providing more medications to treat diabetes and hypertension in this underserved population, instituting screening programs in SRCs with different patient makeups, and determining what type of further care these patients need to prevent further long-term sequelae. This study is unique in that it assesses how demographic factors impact chronic disease presentations in an underserved population. By performing similar research within other SRCs, volunteers can gain deeper insights into the ways in which they can better serve their populations. In future studies, data on socioeconomic factors, health insurance status, and health literacy can also be collected to ensure that they do not serve as confounding variables when assessing how age, race, gender, and ethnicity impact presenting conditions or complaints.
